# Practice Motions Performed During Preperformance Preparation Drive the Actual Motion of Golf Putting

**DOI:** 10.3389/fpsyg.2020.00513

**Published:** 2020-03-25

**Authors:** Yumiko Hasegawa, Akito Miura, Keisuke Fujii

**Affiliations:** ^1^Faculty of Humanities and Social Sciences, Iwate University, Morioka, Japan; ^2^Faculty of Human Sciences, Waseda University, Tokorozawa, Japan; ^3^Graduate School of Informatics, Nagoya University, Nagoya, Japan

**Keywords:** fine motor control, preperformance routine, practice stroke, kinematics, accuracy

## Abstract

Of the various types of preperformance preparatory behavior that are acquired during motor learning, the effect of a practice motion performed just prior to execution of an actual motion is not yet fully understood. Thus, the present study employed a golf putting task to investigate how a practice motion in the preparation phase would affect the accuracy of motor control in the execution phase and how proficiency would influence this relationship. To examine the impacts on kinematics and final ball position, the velocities of practice strokes made by tour professional and amateur golfers were experimentally manipulated in the following three conditions: the equal condition, which presented a target that was at the same distance during the practice strokes and the actual stroke; the confusing condition, which had two different distances during the practice and actual strokes; and the no condition, which did not include a practice stroke. The results, based on final ball position, indicated that practice strokes in the equal condition were linked with the highest accuracy levels during the actual stroke in both professionals and amateurs. In the confusing condition, regardless of skill level, the velocity of the actual stroke was influenced by a faster or slower stroke during the pre-shot phase. These relationships between the practice and actual strokes imply that the golfers effectively utilized kinesthetic information obtained during the practice strokes as a reference for the actual stroke. Furthermore, the differences in proficiency level indicated that the club head velocity of amateurs in the no condition was significantly faster than in the equal condition. Therefore, the present results imply that the role of a practice stroke may differ between professionals and amateurs.

## Introduction

Humans can acquire various skills through learning. For example, if an individual repeatedly practices a motor task, their behavior becomes more accurate (e.g., Crossman, 1959; Proteau et al., 1992) and patterned through the learning process, and acquires consistency (e.g., Magill, 1989; Dhawale et al., 2017). However, according to recent research on skill acquisition, skill learning often has high specificity (Rosenbaum, 2010). For example, studies of the relationship between shooting distance and success rate in basketball players have clarified the role of ‘specificity of practice’ (or ‘specificity of training’), and showed that the success rate at a specific distance (foul line) is higher than would be expected based on the best-fitting line (Keetch et al., 2005). Additionally, assessments of human motor skills when attempting to achieve a target, such as exploring the environment, have demonstrated that behaviors performed just before a trial and practice motions for a trial are just as important as the execution of the movement. For example, quiet eyes (Vickers, 2007), a preparatory body state before reacting (Fujii et al., 2015a, b), and the stepping movement of hitting (Takamido et al., 2019) are improved by practice. Of the various types of preperformance preparation that focus on the practice motion of a motor task, the present study investigated specificity of training in the relationship between a motor skill (golf putting) during the preparation phase (practice strokes) and accuracy of movement during the execution phase (actual stroke). The present findings will further the current understanding of human motor control as well as that of preperformance routines, which are often discussed in sports psychology.

From the perspective of motor control, the ability to adjust muscle strength precisely is a very important factor for various movements (e.g., Fujii et al., 2009; Furuya et al., 2009; Spraker et al., 2009; Kato et al., 2014). In particular, it is difficult for humans to apply small amounts of force precisely during motor control due to the challenging nature of applying the requisite degree of fine motor control. For example, previous studies of hand and fingertip force exertion have shown that the coefficient of variation of the maximal voluntary contraction during low-intensity muscle activity of 10% or less exceeds that in muscle activity of 10% or more (Galganski et al., 1993; Enoka et al., 1999). However, individuals who have trained for a long time and who specialize in a specific task can fine-tune their force output (e.g., Inui and Ichihara, 2001; Kinoshita et al., 2007; Hasegawa et al., 2017). Motor learning is also associated with systematic changes in proprioception (e.g., Haith et al., 2008; Ostry et al., 2010; Wong et al., 2012) such that learning to generate accurate movements improves sensory acuity (Wong et al., 2011). Therefore, experts exhibit a finer degree of kinesthesia compared to non-experts (e.g., Kusanagi et al., 2017; Tanaka and Iwami, 2018). Kinesthesia is an integrated sense of position and limb movement consisting of proprioceptor (muscle and tendon), joint, and cutaneous inputs (Gandevia, 1996). Experts are also able to fine-tune their force output because their recall and recognition schemes are refined (Schmidt, 1975). They also found that long-term training can refine schemas and result in more appropriate parameter settings regarding force, magnitude, and duration.

There are various types and methods of preperformance preparation. In sports, the patterned behaviors performed prior to execution skills that are established by long-term learning are known as preperformance routines and are related to skill expertise. The task success rates of individuals who exhibit consistent preparatory action patterns and movement times are higher than those of individuals who do not (e.g., Wrisberg and Pein, 1992; Lonsdale and Tam, 2008). This is especially true for skill sports, in which performers who use routines have higher success rates than do players who do not (e.g., Lidor and Mayan, 2005; Bell et al., 2010). Accordingly, Cohn (1990) suggested that consistent preparatory actions before task execution effectively activate the recall scheme used for executing a task and that preperformance routines contribute to subsequent accurate movements because “preperformance routines would be to assist the player in selecting a motor program from similar stored responses and to define the specific parameters that will achieve the forthcoming motor response.” Although several studies have investigated preperformance routines, we do not yet have a detailed understanding of how these practice movements typically transfer effective motor paths for motor control to the execution phase.

Golf putting is an appropriate task for examining these problems because this action is a discrete motor skill with a clear beginning and end that is performed using the practice motions in actual game situations. It is based on fine motor skills (Tanaka and Sekiya, 2006) that require the perception of subtle differences in the environment, such as slope and distance to the target, and the fine-tuning of one’s force according to these environmental differences. When putting, it is necessary to control the movement of the club head at a low velocity to achieve high accuracy and reproducibility (Hasegawa et al., 2017). Most golfers perform practice strokes before they hit a ball, which implies that practice strokes have some merit for enhancing the accuracy of their actual stroke. However, the manner in which practice strokes are related to actual strokes remains unclear.

A previous study investigated differences between the practice and actual strokes of professionals and high-level amateurs and how their practice strokes varied according to putting distance (Hasegawa et al., 2019). The authors reported that even highly skilled professional golfers exhibit dramatic differences in the motor control of practice strokes before hitting the ball versus the motor control of the actual stroke according to task constraints, such as the presence or absence of the ball during the stroke. However, for most of these professional golfers, there was excellent correspondence between the club head velocity during the practice phase and that during the actual hitting phase for targets that changed in small steps (0.3 m). In fact, there is a high correlation between actual club head velocity and the distance of ball rolling (Hume et al., 2005). Taken together, professional golfers skillfully respond to subtle changes in target distance during their practice strokes.

Thus, the present research questions were whether practice strokes would contribute to enhancing the accuracy of an actual stroke and whether skill level would affect the relationship between the practice strokes and actual strokes. To answer these questions, the three following conditions were constructed: the equal condition, which presented a target that was at the same distance during the practice strokes and the actual stroke; the confusing condition, which had two different distances during the practice and actual strokes; and the no condition, which did not include a practice stroke. It was predicted that if the practice stroke contributed to enhancing the accuracy of an actual stroke, then performance would be better under the equal condition than under the other conditions (Hypothesis 1). Additionally, if skill level influenced the relationship between the practice strokes and actual strokes, then this relationship would differ between professional and amateur golfers among the conditions (Hypothesis 2). For example, professionals may exhibit a close relationship between practice strokes and actual strokes and, therefore, the velocities of the actual strokes might be more similar to the velocities of the practice strokes in the confusing condition.

Furthermore, the possibility that the effects of skill level would depend on putting distance was considered because differences in skill levels become more apparent at longer distances (Hasegawa et al., 2017, 2019). Therefore, professional and amateur golfers were recruited to investigate the effects of degree of proficiency, condition, and putting distance on club head kinematics, which are key variables involved in the motor control of putting in terms of impact velocity, acceleration profile, and club face angle (Delay et al., 1997; Hume et al., 2005; Karlsen et al., 2008; Dias and Couceiro, 2015).

## Materials and Methods

### Participants

This study included 10 professional tour and 11 amateur golfers with average ages of 31.7 ± 4.4 years and 48.0 ± 11.5 years, respectively, and average experience levels of 19.9 ± 5.3 years and 16.0 ± 9.2 years, respectively. The amateurs were intermediate players with an average handicap of 14.5 ± 1.4. Prior to participation, all participants provided written informed consent after a complete explanation of the study. All experimental procedures were approved by the ethics committee of Iwate University and conformed to the principles of the Declaration of Helsinki.

### Task and Apparatus

The task included two putting distances (1.2 and 7.2 m) under each of the equal, confusing, and no conditions. The two distances were chosen according to the results of a preliminary experiment in which the magnitude (strength) of the practice stroke was significantly different. The putting targets in the equal condition were at the same distance for the practice strokes and the actual strokes, whereas in the confusing condition the putting targets were at two different distances during the practice strokes and the actual strokes. In other words, in the confusing condition, the 7.2-m (1.2-m) target was presented in the practice stroke phase and the 1.2-m (7.2-m) target was presented in the actual hitting phase. In the no condition, participants were asked to perform their actual stroke without making practice strokes. Thus, there was a total of six patterns based on the combinations of conditions and distances, i.e., practice 1.2 m to actual 1.2 m, practice 7.2 m to actual 7.2 m, practice 1.2 m to actual 7.2 m, practice 7.2 m to actual 1.2 m, no to actual 1.2 m, and no to actual 7.2 m. These patterns were presented to each participant in a random order within a set, which included a total of 60 putts that consisted of six patterns and ten sets; no distance information was explicitly conveyed to the participants.

**FIGURE 1:**
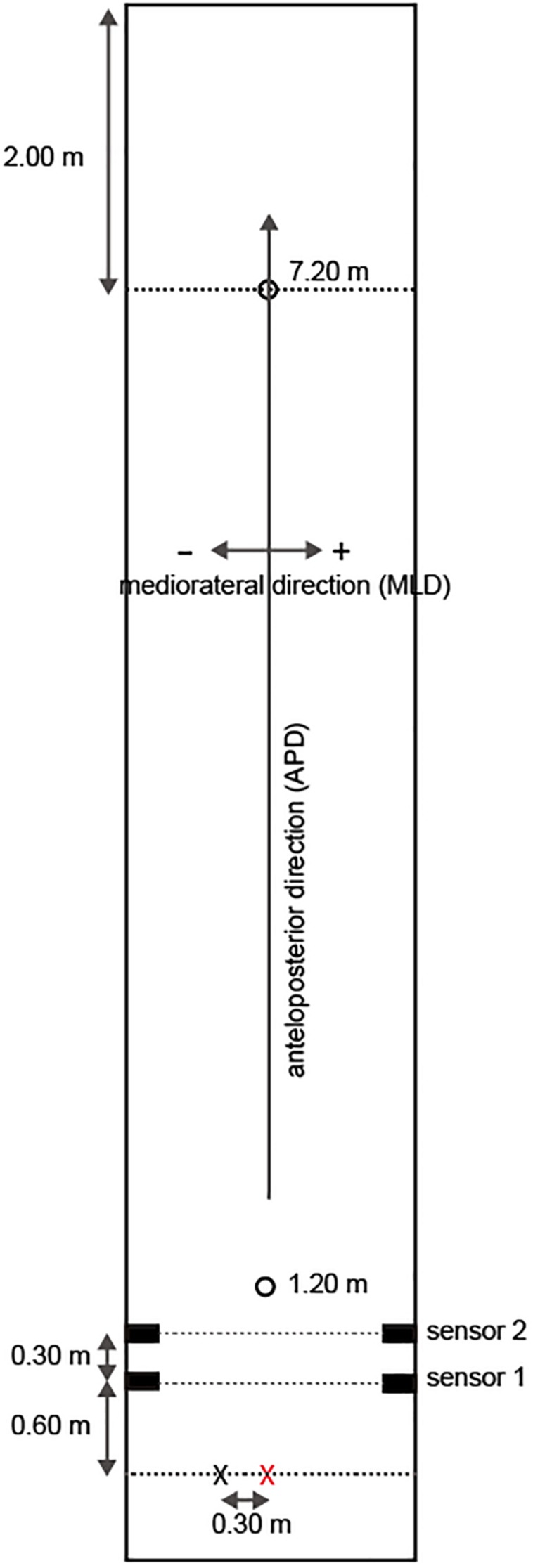
Putting platform. The red cross indicates the point from which the balls were hit and the black cross indicates the virtual ball positions during practice strokes. The participants performed practice strokes as if they intended to hit the presented target at the black cross point. Sensor 1 and Sensor 2 were photoelectric sensors used to measure the initial velocity of a ball.

**FIGURE 2:**
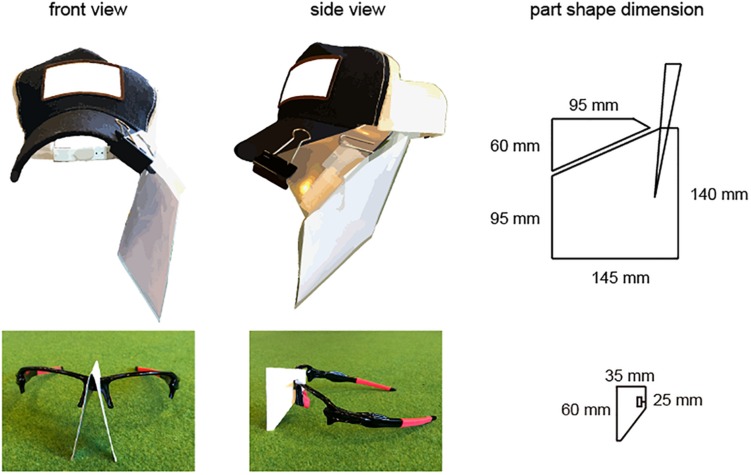
Depiction of the cap and glasses used to limit participants’ fields of vision. All participants wore a cap and sunglasses to limit their field of vision. The cap was fitted with a plastic sheet that was adjusted with two clips, the lenses were removed from the glasses, and a sheet of paper was attached to the nose part of the glasses. The position of the plastic sheet on the cap was adjusted so that the visual field in the hitting direction was 35 cm.

The goal for each participant was to stop the ball in the center of a light beam that was the size of a real hole (10.8 cm in diameter) and was projected using an Offilio EB-1776W ceiling-mounted projector (Epson Corporation; Nagano, Japan) onto a single stretched (9.80 m long × 1.82 m wide; Figure 1) layer of artificial turf that was designed for putting practice (Superbent, Newtons Inc.; Kochi, Japan). The participants were required to perform practice strokes in the pre-shot phase before hitting the ball. When the participants were performing the practice strokes, they were asked to execute the strokes with the intention of hitting the presented target and to finish their practice movements with a final stroke that they felt “made the most appropriate stroke to the presented target.” These guidelines were presented because the purposes of executing practice strokes are diverse and the characteristics of such strokes differ among individuals (Hasegawa et al., 2019). Additionally, the participants wore a cap and glasses (Figure 2) that limited their field of view to provide a 35-cm field ahead of the ball if the participants did not rotate their heads in the direction of a target.

Club head kinematics and final ball positions (FBPs) were recorded with nine optical motion-capture cameras (OptiTrack Prime13, Acuity Inc.; Tokyo, Japan) operating at 240 Hz. Additionally, 12-mm markers were attached to the toe, heel, and neck of the putter head to digitize the positions of the club. The root mean square errors of both the static and dynamic calibrations were <0.5 mm in the range of play (practice and actual stroke areas) whereas they were <1.5 mm in other locations (FBP) during all sessions. All participants used the same putter (SB-01HB, PRGR Corp.; Yokohama, Japan) and balls (Srixon Z-Star XV, Dunlop Sports Co., Ltd.; Hyogo, Japan).

The initial velocity of each ball was measured using two regressive reflection-type photoelectric sensors set at 0.3-m intervals that captured the balls after they were hit by the participants. The distance from the ball hit point to the first sensor was 0.6 m (Figure 1). When a ball crossed the light between the photoelectric sensors, a +5 V signal (TTL signal) was output and then the output signals between sensor 1 and sensor 2 were measured at 2,000-Hz sampling using a Measurement Computing 12-bit A/D Converter. A program using the Dasylab Ver.2016 data acquisition software (National Instruments; Austin, TX, United States) calculated the initial velocity in all trials based on the set distance (i.e., 0.3 m).

### Procedure

In each trial, the participants were told that the practice strokes would be captured and that there were no limits in terms of the number of practice strokes that could be performed or the number of target confirmations prior to the start of the actual stroke. However, after hitting the ball, the participants were not allowed to turn their heads in the direction of the target to confirm the FBP. The participants were also instructed to turn in the opposite direction to the target after the end of their follow-through of the actual stroke and to wait for the next signal from an experimenter. In other words, the participants did not receive visual feedback regarding their performance in any of the trials. Additionally, the participants wore a cap and glasses that restricted their vision. An experimenter adjusted the range of the field of vision so that it was 35 cm ahead of a ball. This range was checked on occasions when the trial was paused (i.e., at breaks). The participants’ hearing was not obstructed. Therefore, they could hear the sound of the impact of the ball. However, in this study, participants could not hear the sound of the ball rolling due to the use of artificial turf, designed for putting.

All participants practiced on the green by executing 24 putts in a predetermined order: six putts at 1.2 m and six putts at 7.2 m × 2, or six putts at 7.2 m and six putts at 1.2 m × 2, including practice strokes in the pre-shot phase; visual confirmation of FBP was acceptable in this phase. Next, the participants received the following instructions: “There are no limits on the number of practice strokes and no limits on confirming the target until the start of actual hitting. In actual trials, there will be cases when the target presented for the actual hit will be different from the target when you practiced in the pre-shot phase. At that time, you need to continue the play and not redo practice strokes. You don’t need to rush, but don’t interrupt the play. Also, if the experimenter guides you ‘Do not practice’ at the beginning of the trial, you need to putt the presented target without practicing.”

The experimenter gave participants a cue to start every trial and also presented a target for the practice strokes. In the equal and confusing conditions, the experimenter erased the target after the practice strokes and then immediately presented the target for the actual stroke. In the no condition, the experimenter called out “Do not practice” and presented a target when they were moving to setup. Following these instructions, each participant made an additional six putts (one for each pattern in a random order) in accordance with the actual trials. In these trials, the participants could not confirm FBP. Each participant completed the abovementioned experimental procedure and was allowed 5-min breaks after putt numbers 18, 30, and 48. The experimenters confirmed whether the participants did or did not turn their heads in the hitting direction after ball impact in all trials.

### Dependent Variables

#### Kinematics

All digitized data were smoothed with a fourth-order Butterworth filter (5-Hz cut-off) based on the root mean square of the residual error between the original and smoothed data (Jackson, 1979; Winter, 1990). The putting movement was divided into the backswing, downswing, ball impact, and follow-through phases (Couceiro et al., 2013; Dias and Couceiro, 2015). Because the ball-roll distance [i.e., the anteroposterior direction (APD)] is highly dependent on impact velocity (Hume et al., 2005; Mathers and Grealy, 2014), the midpoint between the toe and heel of the club head was calculated to analyze the impact velocity and acceleration profile of the midpoint. Impact velocity was defined as the peak velocity of the club head (Hasegawa et al., 2017, 2019). The angle of the club face to the target was calculated using the abovementioned three 12-mm markers (i.e., toe, heel, and neck) because the error of the FBP in the mediolateral direction (MLD) is primarily affected by the face angle (Karlsen et al., 2008). The face angle opens and closes in the MLD during a stroke and, thus, it is slightly more open immediately prior to impact (within 0.005 s) than immediately after impact and differs from the ball’s actual angle of launch (Karlsen et al., 2008).

**FIGURE 3:**
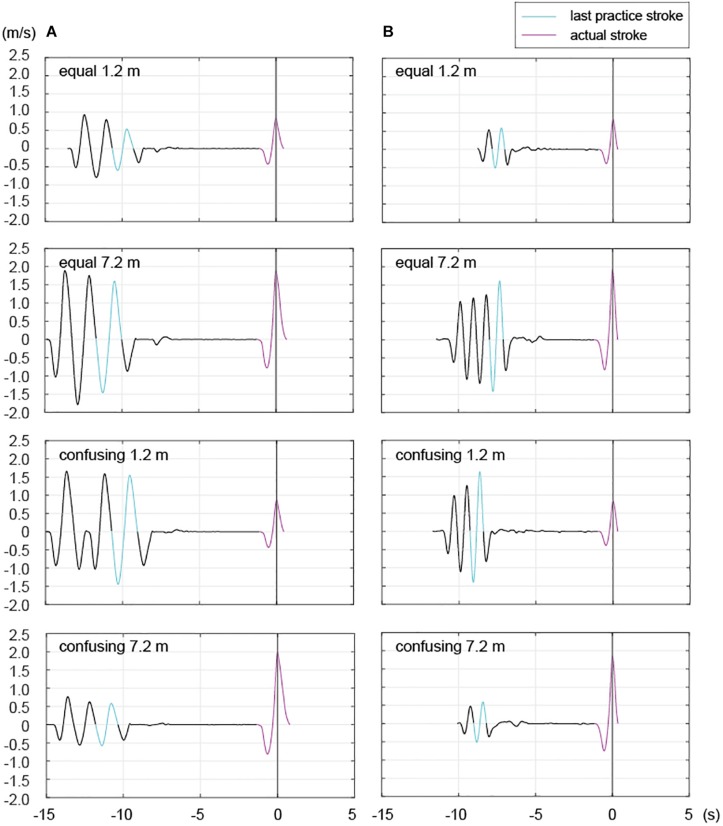
Representative examples of putting strokes for two participants in the equal and confusing conditions. The panels display velocity profiles of two professionals from the start of the backswing for the practice stroke to the end of the follow-through for the actual stroke in the equal and confusing conditions. Light blue lines: last practice stroke; pink lines: actual stroke. The black line between the end of the light blue line and the start of the pink line indicates setting time and Time 0 is the time of impact. As seen in Participant **(A,B)**, the number of practice strokes within an individual varied by trial, as previously reported (Hasegawa et al., 2019).

**FIGURE 4:**
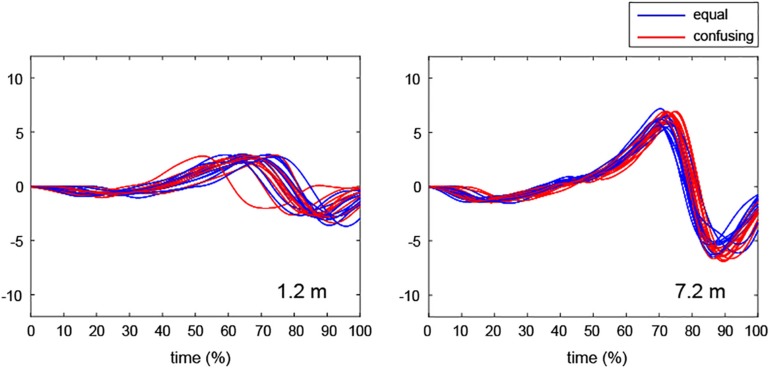
Representative examples of normalized acceleration time scales from the start of the backswing to the end of the follow-through during practice strokes in the equal and confusing conditions. The profiles display all trials of a single professional golfer in the equal condition (blue line) and the confusing condition (red line).

The number of practice strokes performed varies from one to several among different individuals (Hasegawa et al., 2019). In the present study, most participants in most trials performed several practice strokes (average of all: 2.68, standard deviation: 0.71) prior to the actual stroke. The motions of the golfers who made several practice strokes were continuous and, thus, the final practice stroke was analyzed because the participants were asked to finish their practice strokes with the optimal stroke for the target distance (see section “Task and Apparatus”). The impact point of the final practice stroke was defined as just beyond the position where the participant placed the club head prior to commencement of the practice stroke. Thus, the presumed impact point was defined as the real impact point of the practice strokes, as previously described (Hasegawa et al., 2019). Additionally, the start of the backswing for the final stroke was defined as the point where the velocity curve first crossed zero going back from the predicted impact point because this reflects the direction opposite to the hitting direction (where the value of the velocity profile is negative; Figure 3). The present study also analyzed acceleration, which involves a motor control process that imparts appropriate impact velocity. The acceleration time scale was normalized among trials and then the time to peak acceleration was calculated to explore force-control timing during practice strokes (Figure 4).

#### Setting Time

The present study also explored setting time, which was defined as the time from the end of the follow-through of the practice stroke to the start of the actual stroke, in the equal and confusing conditions (Figure 3).

#### Evaluation of Final Ball Position

In the present study, FBP errors were analyzed in terms of APD and MLD and the constant error (CE), variable error (VE), and absolute error (AE) values were determined. When the ball stopped in the center of the light beam (the ‘hole’), the APD and MLD errors were zero. To investigate FBP in further detail, it was divided into three areas each for APD and MLD: less than the target (APD < 0; undershoot), from the center of the target to less than 0.4 m (0 ≤ APD < 0.4 m), and extreme overshooting (0.4 m ≤ APD) and less than 0.2 m to the left or right from the center of the target (−0.2 m < MLD < 0.2 m), more to the right of the target (0.2 ≤ MLD), and more to the left of the target (−0.2 ≥ MLD). The 0.4-m standard was based on Pelz (1989), who found that the optimum ball roll speed to increase the probability of a hole-in is 0.4 m over the hole. Next, the frequency for each of the six areas was calculated. Previous studies have shown that professionals tend to overshoot for long-term training (Hasegawa et al., 2017; Suzuki et al., 2019) and that + 0.4 m overshooting is generalized among golfers (Hasegawa et al., 2017; Suzuki et al., 2019). In other words, if the FBP is within + 0.4 m from the hole, golfers consider that it was a good putt. Furthermore, most golfers are reluctant to make a putt in which the ball does not reach the hole because there is no chance for a hole-in if the ball does not go far enough.

As mentioned above, the size of the putting platform was limited (Figure 1). In particular, for APD, there was a margin of 2.0 m behind the 7.2-m hole such that if the ball exceeded this limit then the FBP could not be determined. Thus, FBP was estimated using the measured initial velocity of the ball for all trials (see section “Task and Apparatus”). Then, the dynamic friction coefficient (μ) was calculated from the initial velocity (*v*), the ball roll distance for APD (*d*), and gravity acceleration (*g* = 9.8 m/s^2^) for each trial as follows:

$$\mu =\frac{v^{2}}{2\,d\,g}$$

For trials in which the FBP could not be measured, errors in the APD of the FBP were calculated using the following formula: $d=\frac{v^{2}}{2\,d\,g}$. μ was computed from the trial in which the initial velocity was closest to the trial for each individual. Errors in the MLD of the FBP that could not be measured were treated as missing values because using the face angle did not allow for proper estimations of the error.

### Statistics

To investigate differences in the average impact velocity between the practice and actual strokes in the equal condition, the relationships among the two groups (professionals and amateurs), two types of strokes (practice and actual), and two distances (1.2 and 7.2 m) were assessed using a three-factor mixed-design analysis of variance (ANOVA). Additionally, the performances of the practice strokes were verified in the equal and confusing conditions. In this experimental procedure, if there is a significant difference between the practice strokes in the equal and confusing conditions, then the validity of the experimental setting cannot be confirmed. Furthermore, if there are no significant differences between the 1.2- and 7.2-m practice strokes in both conditions, then the validity of the experimental setting cannot be confirmed. Thus, the relationships among the groups, conditions (equal and confusing), and distances in terms of average impact velocity, peak acceleration, and time to peak acceleration of the practice strokes were examined with a three-factor mixed-design ANOVA.

Next, the actual strokes in the three conditions were analyzed. Specifically, the relationships among the groups, three types of conditions (practice, actual, and no), and distances in terms of the average impact velocity and face angle of the actual strokes were examined with a three-factor mixed-design ANOVA. Additionally, a three-factor mixed-design ANOVA was conducted to assess differences in the average setting time among the groups, conditions (equal and confusing), and distances. For the FBP, the relationships among the groups, conditions, and distances for the average CE, VE, and AE values for APD and MLD were assessed with mixed-design three-factor ANOVAs. Finally, to assess FBP in more detail, the frequencies for FBP per area were calculated and Chi-squared tests were conducted to compare the three conditions within each group and for each distance.

The results of the three-way ANOVAs are described as follows: second-order interactions (group × condition [or stroke] × distance); first-order interactions (group × condition [or stroke], group × distance, and condition [or stroke] × distance); and main effects. Because putting distance and condition (or stroke) were repeated measures factors, the Bonferroni method was used to account for multiple comparisons. This study also calculated ‘*f*’ values as effect size indices for the ANOVAs and ω values as effect size indices for the Chi-squared tests (Faul et al., 2007). According to the conventions of Cohen (1988), small (*f* = 0.10), medium (*f* = 0.25), and large (*f* = 0.40) effect sizes were reported. All data were analyzed using the PASW Statistics program (ver. 18.0, IBM Japan, Ltd.; Tokyo, Japan).

## Results

### Comparisons of the Practice and Actual Strokes in the Equal Condition

**FIGURE 5:**
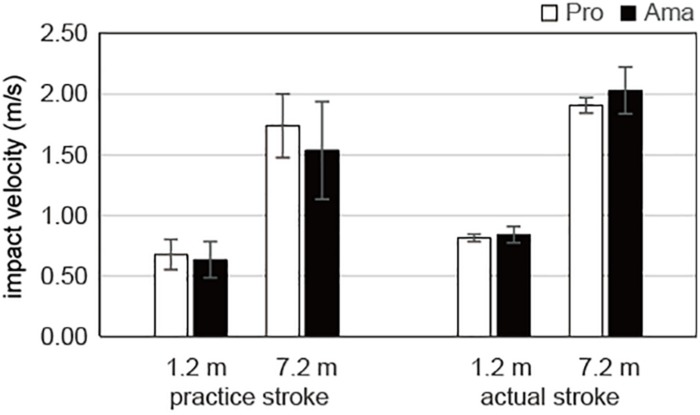
Average impact velocities of the practice and actual strokes in the equal condition. Vertical bars represent the standard deviations for between-subject comparisons.

The results of the three-way ANOVA for impact velocity in the equal condition (Figure 5) revealed that the second-order interaction (group × stroke × distance) was not significant. However, there was a significant first-order interaction (stroke × distance), *F*_1_,_19_ = 6.79, *P* = 0.017, *f* = 0.69. Additionally, simple-effects testing indicated that the impact velocities of the practice strokes for both the 1.2-m distance, *F*_1_,_19_ = 29.56, *P* < 0.001, *f* = 1.24, and the 7.2-m distance, *F*_1_,_19_ = 15.96, *P* = 0.001, *f* = 0.92, were lower than those of the actual strokes and that the impact velocities of both the practice strokes, *F*_1_,_19_ = 333.41, *P* < 0.001, *f* = 4.19, and the actual strokes, *F*_1_,_19_ = 1417.67, *P* < 0.001, *f* = 8.64, differed significantly between the 1.2- and 7.2-m distances. Furthermore, there were significant main effects of stroke, *F*_1_,_19_ = 21.08, *P* = 1.99 × 10^–4^, *f* = 1.05, and distance, *F*_1_,_19_ = 1165.69, *P* = 1.62 × 10^–18^, *f* = 1.05, but not group.

### Comparisons of Practice Strokes in the Equal and Confusing Conditions

**FIGURE 6:**
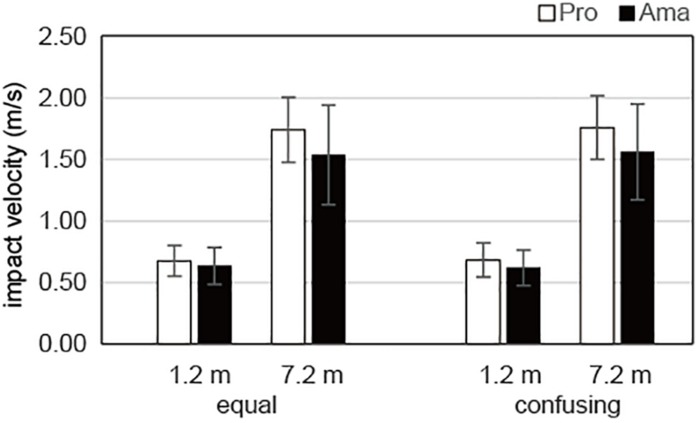
Average impact velocities of the practice strokes in the equal and the confusing conditions. Vertical bars represent the standard deviations for between-subject comparisons.

The results of the three-way ANOVA for the impact velocities of the practice strokes (Figure 6) revealed that there was no significant second-order interaction (group × condition × distance) or first-order interaction (group × condition, group × distance, and condition × distance). Although there was a significant main effect of distance, *F*_1_,_19_ = 336.88, *P* = 1.51 × 10^–13^, *f* = 4.21, no other significant main effects were revealed. In other words, there was a significant difference in impact velocity between the 1.2- and 7.2-m distances, whereas there were no significant differences for practice strokes among the groups and conditions.

**FIGURE 7:**
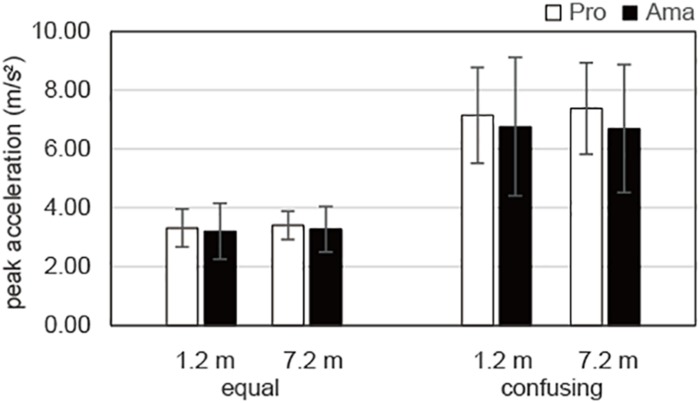
Average peak accelerations of the practice strokes in the equal and the confusing conditions. Vertical bars represent the standard deviations for between-subjects comparisons.

The results of the three-way ANOVA for peak acceleration of the practice strokes (Figure 7) revealed that the second-order interaction and first-order interaction were not significant. Although there was a significant main effect of distance, *F*_1_,_19_ = 122.54, *P* = 1.00 × 10^–10^, *f* = 2.54, there were no other significant main effects. In other words, there was a significant difference in peak acceleration between the 1.2- and 7.2-m distances, whereas there were no significant differences for practice strokes among the groups and conditions.

The results of the three-way ANOVA for time to peak acceleration in the practice strokes (Table 1) revealed that the second-order interaction and first-order interaction were not significant. Although there was a significant main effect of distance, *F*_1_,_19_ = 22.46, *P* = 1.43 × 10^–4^, *f* = 1.09, there were no other significant main effects. In other words, there was a significant difference in the time to peak acceleration between the 1.2- and 7.2-m distances whereas there were no significant differences for the practice strokes among the groups and conditions.

**TABLE 1:** Average times to peak acceleration of the practice strokes in the equal and confusing conditions.

	Equal		Change	
		
	1.2 m	7.2 m	1.2 m	7.2 m
Pro	55.84 ± 5.42	58.56 ± 5.46	54.88 ± 4.33	59.20 ± 6.57
Ama	58.85 ± 5.40	60.46 ± 5.76	57.79 ± 5.68	61.77 ± 5.31

**The numbers after the “±” sign indicate the standard deviation between individuals. Pro, professionals; ama, amateurs; equal, equal condition; change, the change condition.**

### Comparisons of the Three Conditions for Actual Strokes

**FIGURE 8:**
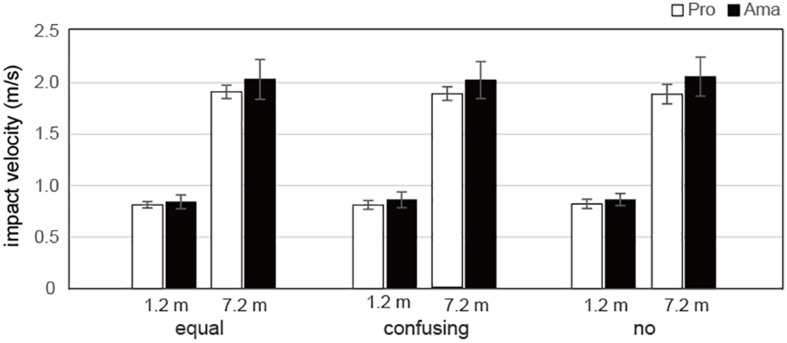
Average impact velocities of actual strokes in the equal, confusing, and no conditions. Vertical bars represent the standard deviations for between-subject comparisons.

The results of the three-way ANOVA for the impact velocity of the actual strokes (Figure 8) revealed that the second-order interactions (group × condition × distance) were not significant whereas the first-order interactions (group × condition) were significant, *F*_2_,_38_ = 3.35, *P* = 0.046, *f* = 0.42. Simple-effects testing indicated that the professionals had lower impact velocities than those of the amateurs in the confusing condition, *F*_1_,_19_ = 5.68, *P* = 0.028, *f* = 0.55, and no condition, *F*_1_,_19_ = 8.23, *P* = 0.010, *f* = 0.66. Additionally, the impact velocities of amateurs varied according to condition, *F*_2_,_38_ = 3.78, *P* = 0.027, *f* = 0.45, and the impact velocities were higher in the no condition than in the equal condition. Moreover, the first-order interactions (condition × distance) were significant, *F*_1_,_19_ = 3.88, *P* = 0.029, *f* = 0.45. Simple-effects testing indicated that there were significant differences in impact velocities between the 1.2- and 7.2-m distances in the equal, *F*_1_,_19_ = 1420.21, *P* < 0.001, *f* = 8.65, confusing, *F*_1_,_19_ = 1540.34, *P* < 0.001, *f* = 9.00, and no, *F*_1_,_19_ = 1308.02, *P* < 0.001, *f* = 8.30, conditions. There were also significant main effects of group, *F*_1_,_19_ = 6.14, *P* = 0.023, *f* = 0.57, and distance, *F*_1_,_19_ = 1549.81, *P* = 1.12 × 10^–19^, *f* = 9.03.

The results of the three-way ANOVA for the face angles of actual strokes (Supplementary Table S1) revealed that the second-order interactions and first-order interactions were not significant. Likewise, there were no significant main effects.

### Setting Time

The results of the three-way ANOVA for setting time (i.e., from the end of the practice stroke to the start of the actual stroke; Table 2) revealed that the second-order interactions and first-order interactions were not significant. However, there were significant main effects of condition, *F*_1_,_19_ = 26.26, *P* = 6.02 × 10^–5^, *f* = 1.18, and distance, *F*_1_,_19_ = 13.80, *P* = 0.001, *f* = 0.85. In other words, setting times were longer in the confusing condition than in the equal condition and were shorter for the 1.2-m distance than for the 7.2-m distance, regardless of group.

**TABLE 2:** Average setting times in the equal and confusing conditions.

	Equal		Confusing	
		
	1.2 m	7.2 m	1.2 m	7.2 m
Pro	8.15 ± 1.60	9.38 ± 2.22	9.31 ± 2.58	10.44 ± 3.53
Ama	8.92 ± 3.36	10.08 ± 3.88	9.88 ± 3.63	10.59 ± 3.79

**The numbers after the “±” sign indicate the standard deviation between individuals. Pro, professionals; ama, amateurs; equal, equal condition; confusing, the confusing condition.**

### FBP Assessments

**FIGURE 9:**
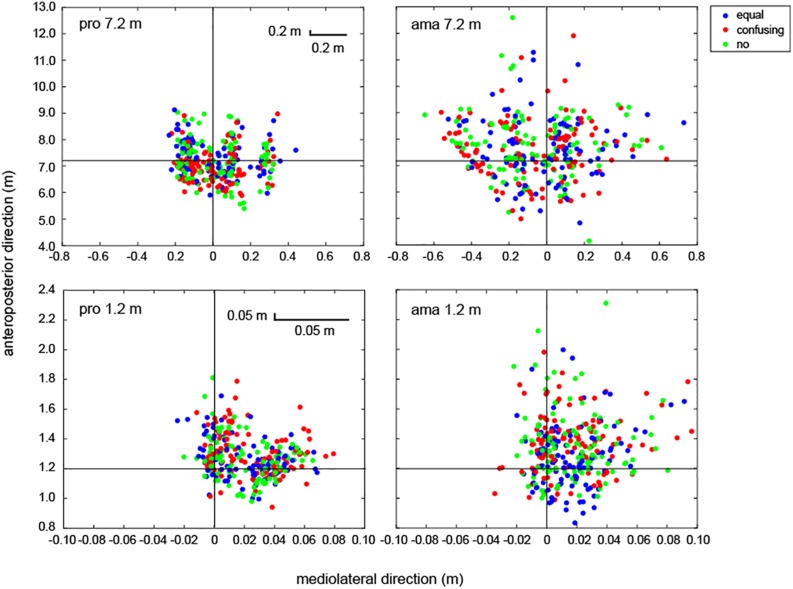
All FBPs of the professional and amateur golfers. The point at which the black horizontal and vertical lines cross indicates the center of the target. Circles are indicative of the condition: equal condition = blue, confusing condition = red, and no condition = light green. The **upper-left panel** shows all putting of professionals at 7.2 m (*n* = 10; total of 300 trials). The **upper-right panel** shows all putting of amateurs at 7.2 m (*n* = 11; total of 330 trials). The **lower-left panel** shows all putting of professionals at 1.2 m (a total of 300 trials). The **lower-right panel** shows all putting of amateurs at 1.2 m (total of 330 trials).

Figure 9 shows all putting trials for all participants. The measured values and estimated values of the FBPs were calculated using the dynamic friction coefficient (μ) and were compared in all trials of all participants. The average absolute error between the measured values and estimated values for the 7.2-m distance was 0.25 ± 0.09 m, which was approximately 3.6% of the distance to the target in terms of APD. For the amateur golfers, there was a total of 16 trials (conditions: equal = 6, change = 5, and no = 5; 1–7 trials in each of seven amateurs) in which the ball was hit more than 9.2 m and, thus, was unmeasurable. There were no unmeasurable trials for the professional golfers.

#### CE Assessments

**FIGURE 10:**
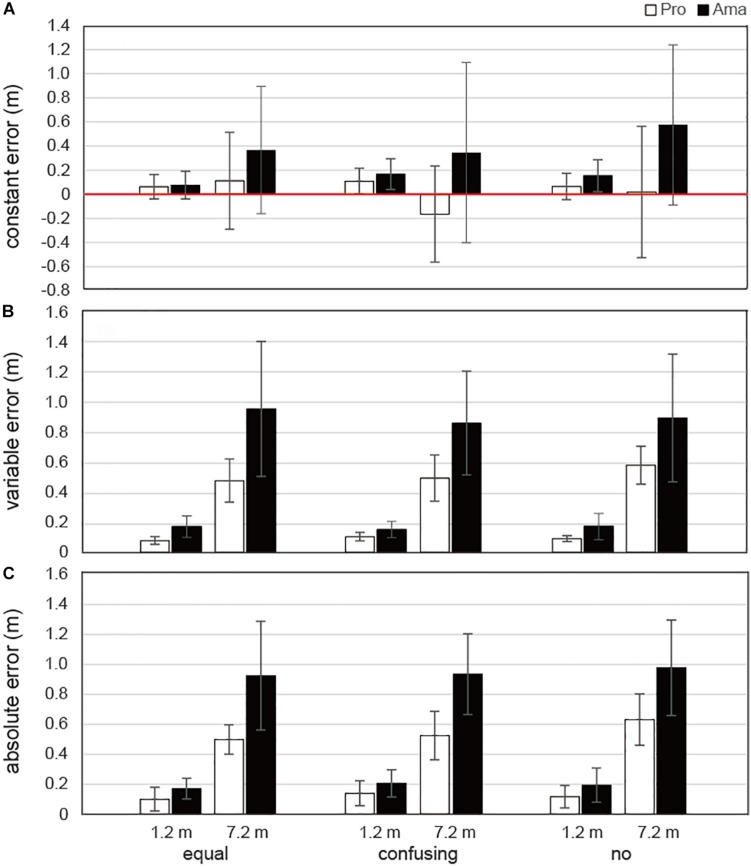
Average errors in FBP for the APD. Panel **(A)** shows the average constant error (CE). Panel **(B)** shows the average variable error (VE). Panel **(C)** shows the average absolute error (AE). Vertical bars represent the standard deviations for between-subject comparisons.

The results of the three-way ANOVA for CE in terms of APD (Figure 10A) revealed that the second-order interactions (group × condition × distance) were not significant whereas there were significant first-order interactions (condition × distance), *F*_2_,_38_ = 4.89, *P* = 0.013, *f* = 0.50. Additionally, there were significant simple main effects of the 1.2-m distance, *F*_2_,_38_ = 9.76, *P* < 0.001, *f* = 0.72, and 7.2-m distance, *F*_2_,_38_ = 3.35, *P* = 0.046, *f* = 0.42. The multiple comparison results indicated that the CE was lower at 1.2 m in the equal condition than in the confusing and no conditions and lower at 7.2 m in the confusing condition than in the no condition. There was also a significant main effect of group, *F*_1_,_19_ = 4.65, *P* = 0.045, *f* = 0.49, which indicated that the CE was lower for professionals than for amateurs. The results of the three-way ANOVA for CE in terms of MLD (Supplementary Table S2) revealed that the second-order interactions, first-order interactions, and main effects were not significant.

#### VE Assessments

The results of the three-way ANOVA for VE in terms of APD (Figure 10B) revealed that the second-order interactions were not significant whereas the first-order interactions (group × distance) were significant, *F*_1_,_19_ = 8.32, *P* = 0.05, *f* = 0.66. Simple-effects testing indicated that the VEs of the professionals were lower than those of the amateurs at both the 1.2-m distance, *F*_1_,_19_ = 12.40, *P* = 0.002, *f* = 0.81, and the 7.2-m distance, *F*_1_,_19_ = 11.65, *P* = 0.003, *f* = 0.78, and that the 1.2-m VEs of both the professionals and amateurs, *F*_1_,_19_ = 29.44, *P* < 0.001, *f* = 1.24, were smaller than their 7.2-m VEs, *F*_1_,_19_ = 97.45, *P* < 0.001, *f* = 2.26. There were also significant main effects of group, *F*_1_,_19_ = 14.35, *P* = 0.001, *f* = 0.87, and distance, *F*_1_,_19_ = 115.32, *P* = 1.65 × 10^–9^, *f* = 2.46.

The results of the three-way ANOVA for VEs in terms of MLD (Supplementary Table S2) revealed that the second-order interactions were not significant whereas the first-order interactions (group × distance) were significant, *F*_1_,_19_ = 13.39, *P* = 0.002, *f* = 0.84. Simple-effects testing indicated that the VEs of the professionals were lower than those of the amateurs at the 7.2-m distance, *F*_1_,_19_ = 14.27, *P* = 0.001, *f* = 0.87, and that the 1.2-m VEs of professionals, *F*_1_,_19_ = 50.65, *P* < 0.001, *f* = 1.63, and amateurs, *F*_1_,_19_ = 162.97, *P* < 0.001, *f* = 2.93, were lower than the 7.2-m VEs. There were also main effects of group, *F*_1_,_19_ = 14.61, *P* = 0.001, *f* = 0.88, and distance, *F*_1_,_19_ = 194.87, *P* = 1.94 × 10^–11^, *f* = 3.21. Additionally, the results revealed that the VEs for professionals in terms of MLD were lower than those of amateurs and that the 1.2-m VEs were lower than the 7.2-m VEs, regardless of condition.

#### AE Assessments

The results of the three-way ANOVA for AE in terms of APD (Figure 10C) revealed that the second-order interactions were not significant whereas the first-order interactions (group × distance) were significant, *F*_1_,_19_ = 9.56, *P* = 0.006, *f* = 0.71. Simple-effects testing indicated that the AEs of professionals were lower than those of amateurs at both the 1.2-m distance, *F*_1_,_19_ = 4.40, *P* = 0.050, *f* = 0.48, and 7.2-m distance, *F*_1_,_19_ = 16.56, *P* = 0.001, *f* = 0.93, and that the 1.2-m AEs of both the professionals and amateurs, *F*_1_,_19_ = 32.50, *P* < 0.001, *f* = 1.31, were smaller than the 7.2-m AEs, *F*_1_,_19_ = 109.42, *P* < 0.001, *f* = 2.40. There were also significant main effects of group, *F*_1_,_19_ = 21.32, *P* = 1.88 × 10^–4^, *f* = 1.06, and distance, *F*_1_,_19_ = 128.69, *P* = 6.65 × 10^–10^, *f* = 2.60.

The results of the three-way ANOVA for AEs in terms of MLD (Supplementary Table S2) revealed that the second-order interactions were not significant whereas the first-order interactions (group × distance) were significant, *F*_1_,_19_ = 5.28, *P* = 0.033, *f* = 0.53. Simple-effects testing indicated that the AEs of the professionals were lower than those of the amateurs at 7.2 m, *F*_1_,_19_ = 5.22, *P* = 0.033, *f* = 0.52, and that the 1.2-m VEs of professionals, *F*_1_,_19_ = 18.23, *P* < 0.001, *f* = 0.98, and amateurs, *F*_1_,_19_ = 60.98, *P* < 0.001, *f* = 1.79, were lower than the 7.2-m VEs. There were also significant main effects of group, *F*_1_,_19_ = 4.80, *P* = 0.041, *f* = 0.88, and distance, *F*_1_,_19_ = 71.90, *P* = 6.99 × 10^–8^, *f* = 1.95. Additionally, the results indicated that the AEs of professionals in terms of MLD were lower than those of amateurs and that the 1.2-m VEs were lower than the 7.2-m VEs, regardless of condition.

#### Frequency of FBP by Area

The APD counts for each group and each distance are displayed in Tables 3, 4, respectively. Chi-squared tests revealed that there was a significant difference in APD among the three conditions only for professionals at the 7.2-m distance (χ[4] = 9.56, *P* < 0.05, ω = 0.18; Table 3). A residual analysis indicated that the number of negative FBPs (i.e., undershooting) was significantly higher in the confusing condition than the other two conditions and that there was a significant decrease in overshooting (0.4 m ≤ APD). Examinations of the MLD revealed no significant differences for distance according to condition in either of the two groups.

**TABLE 3:** Frequency of FBP by area for APD in professional golfers.

	1.2 m			7.2 m		
		
	Equal	Confusing	No	Equal	Confusing	No
APD < 0	37	23	31	51	67 Δ	54
0 ≤ APD < 0.4	62	74	67	16	17	14
0.4 ≤ APD	1	3	2	33	16 ∇	32

**Chi-squared tests were conducted for each distance. FBP, final ball position; equal, equal condition; confusing, the confusing condition; no, the no condition. The triangle marks indicate the results of the residual analysis*.*

**TABLE 4:** Frequency of FBP by area for APD in amateur golfers.

	1.2 m			7.2 m		
		
	Equal	Confusing	No	Equal	Confusing	No
APD < 0	38	25	29	39	43	33
0 ≤ APD < 0.4	63	68	68	20	16	14
0.4 ≤ APD	9	17	13	51	51	63

**Chi-squared tests were conducted for each distance. FBP, final ball position; equal, equal condition; confusing, the confusing condition; no, the no condition*.*

This study also conducted Chi-squared tests of the professionals at the 7.2-m distance as well as additional analyses for undershoots and extreme overshoots (0.4 m ≤ APD) for individuals (Supplementary Table S3). Similar to the results for APD < 0, a two-factor ANOVA that included group (professionals and amateurs) and condition (equal, confusing, and no) revealed no significant interaction effects but significant main effects of group, *F*_1_,_19_ = 4.20, *P* = 0.054, *f* = 0.47, and condition, *F*_2_,_38_ = 3.73, *P* = 0.054, *f* = 0.44. Multiple comparison analyses indicated that the FBP was lower in the confusing condition than in the no condition. For an APD ≥ 0.4 m, the interaction was not significant whereas there were significant main effects of group, *F*_1_,_19_ = 4.89, *P* = 0.039, *f* = 0.51, and condition, *F*_2_,_38_ = 4.39, *P* = 0.019, *f* = 0.48. Multiple comparison analyses indicated that the FBP was lower in the confusing condition than in the no condition.

## Discussion

The present study employed a golf putting task to investigate how practice motions in the preparation phase of a movement would influence the accuracy of motor control during the execution phase and to determine how proficiency would affect this relationship. The practice strokes of golfers were manipulated by the construction of equal, confusing, and no conditions.

First, this study compared practice strokes between the 1.2- and 7.2-m distances in the equal and confusing conditions. The motor control patterns of practice motions and actual motions differed due to variations in the constraints of a task (Marteniuk et al., 1987; Tanaka et al., 2016). The degree of difference varied among individuals because the purposes of practice motions vary among individuals (Hasegawa et al., 2019). Therefore, in this study, the participants were asked to unify their strategy and “make practice strokes with the intention of hitting a presented target.” This allowed an examination of the relationships among proficiency, condition, and distance in terms of impact velocity, maximum acceleration, and time to peak velocity. Analyses of impact velocity in this study revealed that the participants performed similar practice strokes for the same distance in both the equal and confusing conditions regardless of skill level. This finding confirmed the validity of the experimental settings. This study also examined the maximum acceleration and the peak of the acceleration profile to clarify how the kinematics of practice strokes performed at the 1.2- and 7.2-m distances differed. The results revealed that the magnitude of the club head accelerations exerted during the practice strokes for the 7.2-m distance was about twice that of the 1.2-m distance and that the peak acceleration of the 7.2-m distance was delayed compared to that of the 1.2-m distance (Table 1). Taken together, there were no differences between the two conditions whereas the magnitude and timing of the force exerted during swinging differed between the target distances.

This study also assessed how practice strokes would affect the actual stroke in the confusing condition, where the target distances were not congruent. Analyses of the CE values of the FBP in terms of APD confirmed that the CE of professionals was smaller than that of amateurs, as previously reported (Hasegawa et al., 2019). Regardless of skill level, accuracy (CE) was higher at the 1.2-m distance in the equal condition than in the confusing and no conditions. Therefore, at 1.2 m, both professionals and amateurs had the best results when performing appropriate practice strokes, which supports Hypothesis 1 of this study. These results also support the views of Cohn (1990) who showed that preperformance preparatory motions contribute to the setting of the force parameters at the time of the actual performance regardless of skill level. The present study also found that the CE was lower in the confusing condition than in the no condition at the 7.2-m distance, which is in contrast to the present hypothesis predicting that the equal condition would be the most accurate. In the equal condition, there was a tendency to overshoot the target at both the 1.2- and 7.2-m distances, which is a bias that is particularly evident in golf experts and is similar to the findings of previous reports (Hasegawa et al., 2017; Suzuki et al., 2019). Although there were large individual differences in performance at the 7.2-m distance in the confusing condition, the FBPs of the professionals and amateurs appeared to show opposite patterns. Therefore, the 7.2-m results need to be interpreted carefully. However, it was possible to further assess these data by analyzing the frequency of FBPs according to area.

After categorizing FBP frequency according to area, the results showed that only the FBP for professionals at the 7.2-m distance was significantly biased (see Table 3). These results provide a clear indication of whether each putt was a mistake (see detailed information in section “Evaluation of Final Ball Position”) because this means that there was an increase in the number of undershot putts that deviated from the tolerance area (within +40 cm). However, it is possible that an increased number of undershoots from only a few experts could have caused these results and, therefore, FBP frequency according to area was also analyzed in each individual participant (see Supplementary Table S3). The difference in proficiency was not significant as undershoots increased in 70% of the professionals in the confusing condition and 45% of amateurs in the equal condition.

Because the impact velocity of an actual stroke is the major determinant of the ball rolling distance, analyses of this characteristic will detail how practice strokes are involved in a golfer’s motor control. In this study, the impact velocities of professionals were lower than those of amateurs in the confusing and no conditions. In general, humans are not initially adept at precisely controlling small forces (Enoka et al., 1999). The present findings confirmed that the force output of amateurs during an actual stroke was stronger than that of professionals, which supports previous findings showing the poor resolution of motor control by amateurs (Hasegawa et al., 2017). Furthermore, the present study found that the no condition had the greatest adverse effect on the velocity of actual strokes in amateurs. That is, if amateurs did not execute practice strokes, then they may hit the ball harder. This result also implies that there was a skill difference regarding the connection between the practice strokes and actual strokes, which supports Hypothesis 2 of the present study. Similarly, Cohn (1990) reported that preparatory routines operate as a warm-up session following a period of reset, and aid in the return of an appropriate set that facilitates a performance routine. Thus, for amateurs, practice strokes may function as a warm-up that prevents excessively hard hits during actual strokes. The generalizability of our findings requires further supporting data to obtain more reliable results (i.e., more golfers).

The confusing condition in the present study allowed for a better understanding of the role of practice motions during the pre-shot phase. The result that appropriate practice strokes lead to accurate actual hit indicate that practice motions made in the preparatory phase significantly contributed to the setting of parameters used in the actual performance of the putting behavior, as suggested by Cohn (1990) and Hasegawa et al. (2019). Furthermore, the results of the setting time analyses revealed that the average setting time was significantly longer in the confusing condition than in the equal condition (see Table 2), which reflects the difficulty of such tasks (Wilson et al., 2007). Because the distances to the target differed between the practice strokes and actual strokes in the confusing condition, the present participants took longer to plan their motor execution in the confusing condition than in the equal condition. In general, experts are consistently faster and more accurate than novices in a variety of perceptual-cognitive paradigms (Starkes and Ericsson, 2003; Hodges et al., 2006; Mann et al., 2007). Therefore, even in the confusing condition in the present study, it is probable that the professionals could more accurately respond to the task from when they were setting the club head until starting the actual stroke. However, the present results are not consistent with this idea, which may have been due to task characteristics. Golf is a self-paced task and even professional golfers are not required to flexibly change their plans against their intentions. However, the increased numbers of undershot putts by the professionals and amateurs in the confusing condition cannot be fully explained by confusion during movement planning. If that were the only cause, then overshoot errors should also increase. It is possible that the golfers, particularly the professionals, effectively utilized kinesthetic information obtained from the practice strokes in the pre-shot phase as a reference. Further, the result that the velocity of the practice strokes drives the actual stroke may be explained by assimilation effects in discrete movements (Marteniuk et al., 1984; Sherwood, 2007, 2008). Future research should explore this possibility further. The other error indices indicated that the practice strokes in the confusing condition and the lack of practice strokes in the no condition did not significantly influence control of the face angle of the club head (see detail in Supplementary Table S1).

Regarding the kinematics of the swing, the present study found that the practice strokes at the 1.2- and 7.2-m distances differed in the magnitude of acceleration and in the timing of the acceleration. Previous studies that evaluated force control have suggested that the magnitude of prior force may influence the control of one’s posterior force (Laabs, 1973). Although the present results appear to support those of Laabs (1973), a variety of factors, such as the size of the previous force, size of the required level, and size of the change amount, are associated with the history of force control and are involved in this process (e.g., Spiegel et al., 1996; Harbst et al., 2000; Ohtaka and Fujiwara, 2019). It has been shown that changing one’s timing has a greater effect on motor control than changing the magnitude of one’s force (Rinkenauer et al., 2001). However, it remains unclear whether control of a prior force influences control of the posterior force, and the underlying mechanisms could not be elucidated by this study. Although the intentions of the participants were unified by the instructions (“make practice strokes with an intention to hit a presented target”), there were slight differences among the individuals in club head velocity during practice strokes. Therefore, the manner in which individual differences in club head velocity during practice strokes affected the outcome remains unclear. It might be beneficial to capture the club head velocity exerted during practice swings in real-time and have an experimenter control club head velocity at a constant. However, doing this would likely interfere with the preperformance preparations of participants. Thus, future research will be necessary to address these concerns.

## Conclusion

Taken together, the present findings indicate that practice motions contributed to the accuracy of actual motions during golf putting and that it was important to execute the motions at an appropriate velocity to ensure accurate performance for amateurs as well as professionals. The present findings also suggest that practice swing velocity could drive actual swing velocity regardless of skill level. Furthermore, in the present study, the impact velocity of the amateurs was particularly affected by a lack of practice motions and, thus, there was a difference in their proficiency between the practice and actual motions. Taken together, these findings can provide novel insights into how humans achieve fine force control and how this process might be reflected in motor learning during the acquisition of higher-level skills.

## Data Availability Statement

The datasets analyzed for this study can be found in the Supplementary Data Sheet S2.

## Ethics Statement

The studies involving human participants were reviewed and approved by the internal review board of Iwate University. The patients/participants provided their written informed consent to participate in this study.

## Author Contributions

YH, AM, and KF conceived and designed the experiments. YH performed the experiments. YH and KF analyzed the data. All authors contributed to, and have approved, the final manuscript.

## Conflict of Interest

The authors declare that the research was conducted in the absence of any commercial or financial relationships that could be construed as a potential conflict of interest.
