# Supplementary material for: Practice Motions Performed During Preperformance Preparation Drive the Actual Motion of Golf Putting
Source: Front Psychol. 2020 Mar 25;11:513. doi: 10.3389/fpsyg.2020.00513 (PMC7109320; doi:10.3389/fpsyg.2020.00513)
Supplement: Supplementary file 1 [file Data_Sheet_1.pdf]

Supplementary Material

1 Supplementary Tables

Supplementary Table 1. Average impacts of face angle on actual strokes in the three conditions.

		equal		confusing		no	
		1.2 m	7.2 m	1.2 m	7.2 m	1.2 m	7.2 m
pro	average	2.30	2.36	2.48	2.30	2.45	2.44
	sd	1.88	1.64	1.72	1.90	1.41	1.48
ama	average	1.87	1.35	1.85	1.14	1.86	1.31
	sd	1.85	2.12	1.65	1.85	1.67	1.85

Note: Pro: professionals. Ama: amateurs. Equal: the equal condition. Confusing: the confusing condition. No: the no condition. CE: constant error. VE: variable error. AE: absolute error. Sd: standard deviation.

Supplementary Table 2. Average errors in the FBP for MLD.

			equal		confusing		no	
			1.2 m	7.2 m	1.2 m	7.2 m	1.2 m	7.2 m
CE	pro	average	0.020	0.007	0.025	0.004	0.020	0.018
		sd	0.018	0.138	0.019	0.126	0.017	0.130
	ama	average	0.016	-0.031	0.017	-0.054	0.018	-0.070
		sd	0.014	0.191	0.019	0.223	0.017	0.216
VE	pro	average	0.011	0.085	0.012	0.073	0.011	0.080
		sd	0.003	0.034	0.005	0.032	0.005	0.021
	ama	average	0.014	0.138	0.016	0.126	0.015	0.129
		sd	0.006	0.032	0.006	0.041	0.005	0.054
AE	pro	average	0.022	0.135	0.026	0.121	0.022	0.128
		sd	0.015	0.054	0.018	0.060	0.015	0.053
	ama	average	0.019	0.189	0.022	0.209	0.022	0.211
		sd	0.012	0.083	0.015	0.102	0.014	0.107

Note: Pro: professionals. Ama: amateurs. Equal: the equal condition. Confusing: the confusing condition. No: the no condition. CE: constant error. VE: variable error. AE: absolute error. Sd: standard deviation.

Supplementary Table 3. Frequency of FBP by area in each individual at 7.2 m.

	APD < 0 m			0.4 m ≤ APD		
	equal	confusing	no	equal	confusing	no
pro 1	3	5	3	4	1	4
pro 2	4	9	7	4	0	1
pro 3	2	3	3	6	6	5
pro 4	4	6	4	5	0	5
pro 5	5	5	4	4	3	4
pro 6	9	10	10	0	0	0
pro 7	8	7	5	1	2	4
pro 8	10	9	10	0	0	0
pro 9	3	8	7	4	1	2
pro 10	3	5	1	5	3	7
ama 1	3	4	4	5	5	3
ama 2	7	10	8	1	0	2
ama 3	0	1	0	10	7	10
ama 4	5	4	5	5	4	4
ama 5	3	0	2	6	10	7
ama 6	4	9	6	2	0	1
ama 7	5	6	4	2	2	6
ama 8	3	3	3	6	5	7
ama 9	4	4	1	3	4	7
ama 10	0	0	1	9	8	9
ama 11	5	2	0	2	6	6

Note: Cases that increased compared to the equal condition are shown in yellow whereas cases that reduced compared to the equal condition are shown in green.
